# How do medical students value health on the EQ-5D? Evaluation of hypothetical health states compared to the general population

**DOI:** 10.1186/1477-7525-6-111

**Published:** 2008-12-11

**Authors:** Maria-Theresa Barbist, Daniela Renn, Bianca Noisternig, Gerhard Rumpold, Stefan Höfer

**Affiliations:** 1 Clinic of Medical Psychology, Medical University Innsbruck, Schöpfstraße 23a, 6020 Innsbruck, Austria; 2 Oppolzerstr. 8, 6020 Innsbruck, Austria; 3 Öffentliches Landeskrankenhaus Natters, In der Stille 20, 6161 Natters, Austria

## Abstract

**Background:**

Medical students gain a particular perspective on health problems during their medical education. This article describes how medical students value 10 hypothetical health states using the EQ-5D compared to the general population.

**Methods:**

Based on a sample of 161 medical students (male: 41%) we compared valuations of 10 hypothetical EQ-5D health states collected in face to face interviews with the valuations of the general population. Self-reported health on the EQ-5D was also collected.

**Results:**

Every third health state was valuated higher by the medical students compared to data of the general population. The differences were independent of the severity of the hypothetical health state. Concerning the self-reported health, the majority of the students (66%) reported no problems in the five EQ-5D domains (EQ-5D VAS M = 87.3 ± 9.6 SD). However, when compared to an age-matched sample the medical students show significantly more problems in the area of pain/discomfort and anxiety/depression.

**Conclusion:**

Medical students have a tendency to value health states higher than the general public. Medical professionals should be continuously aware that their assessment of the patients health state can differ from the valuations of the general population.

## Background

The assignment of preferences to certain health states is a critical and controversial topic. This is especially true when it comes to valuing our own health in contrast to valuing health of others. In any case people refer to the salient and most important aspects of their own lives to value health states. These valuations can be implicit or explicit, however they always exist. Medical decisions on an individual basis or policy basis are and always will be influenced by these valuations [1].

In a recent European survey on the acceptance of quality of life measurement between 72–90% of the physicians accepted quality of life (QoL) as an outcome measure, however with less than 50% accepting the concept of quality adjusted life years (including utility measurement) [2]. In a similar survey in the United States and Canada only about one third of the physicians had ever collected data on quality of life or had taken it systematically into account in clinical decision making [3]. Therefore it is of importance within the medical curriculum to sensitize students to the impact of QoL and health state valuations on the decision making process by involving them in health valuation tasks. Medical students gain a different perspective on health problems during their medical education by developing the role of a medical doctor. The participation in a health state valuation task potentially allows them to reflect on a patients' perspective on decision making when being confronted with hypothetical health states. Further it has been acknowledged that there is a need for health related quality of life education in medical school [4].

Methods for generating health preferences are based on the development of decision theory. Using health preferences, quality-adjusted life-years (QALYs) can be calculated. Conceptually QALYs summarize the treatment outcome in terms of time spent in a particular health state and with a particular quality of life [5]. Different tools were developed to measure health states or health-related quality of life (HRQL), which allow the calculation of QALYs, e.g. Short Form-6D [6], Health Utility Index I-III [7] and EQ-5D [8].

The EQ-5D is a well established health-utility index measure and was originally designed to complement other forms of quality of life measures. It has been purposefully developed to generate a cardinal index of health, therefore it has considerable potential for use in economic valuation [9]. It is widely used for monitoring the health status of patient groups at different points in time: (1) for valuation and audit of health care, by measuring changes in health status in individual patients and in groups of patients; (2) further for assessing the seriousness of conditions, providing relevant information for resource allocation at a variety of levels; (3) assisting in providing evidence about medical effectiveness in processes where drugs or procedures have to be approved; (4) monitoring and establishing population health status locally, nationally and internationally [8]. The standard approach of the EuroQol group to establish health preferences is the Visual Analogue Scale rating [9].

The purpose of this study was to describe how medical students value 10 hypothetical health states using the EQ-5D in comparison to the general population.

## Methods

In face to face interviews with 180 students of the Medical University Innsbruck, conducted in 2001 and 2002, we collected data on self-reported health and valuations of EQ-5D hypothetical health states. The participation in this study was part of their educational programme during one term in their second year of medical school, that included a basic lecture on quality of life. Participation was voluntary and anonymous. Ethical approval was obtained from the institutional review board.

We used the German version of the EQ-5D for which data of the general population of Germany were available.

The EQ-5D consists of 5 dimensions (mobility, self-care, usual activity, pain/discomfort, anxiety/depression). For each dimension there are three answer categories: no problem (1), some problems (2), or severe problems (3). The combination of five dimensions with three answer categories [3^5^] result in 243 possible health states described as vectors (e.g. 11231, no problems walking around, no problems with self care, some problems with performing usual activities, extreme pain or discomfort and not anxious or depressed). The second component of the EQ-5D is a visual analogue scale (VAS), providing the respondents with the option to describe their current overall health status on a thermometer-type scale ranging from 0 – 100 [8].

A trained interviewer guided the participants in groups of 10 people through the valuation process. First, students rated their own health status by completing both parts of the EQ-5D questionnaire. Second, ten of the possible 243 health states were presented for the valuation task.

We chose 10 hypothetical health states out of a previously used set of health states used for modelling the full set of EQ-5D health states [10]. The set included 2 health states of the category "very mild" (11112, 21111), 2 "mild" health states (11113, 12121), 3 "moderate" health states (12222, 21232, 21323) and 3 "severe" health states (22323, 32223, 32232).

The interviewers asked the respondents to rank the 10 hypothetical health states from best to worst according to their individual perception. In a next step the students marked each health state on a thermometer-style VAS according to its relative rank. The VAS was bounded by the worst imaginable health state (0) and the best imaginable health state (100). Participants were encouraged to use a form of "bisectioning," where they begin by marking the best and worst states on the rating scale followed by the intermediate states.

Since there is no EQ-5D data available for the general population of Austria, we compared our data on self-reported health of medical students with the self-reported health data of a German general population sample [11]. The German sample was randomly selected to create norm values for the general population. 2022 subjects between 19 and 93 years of age were tested with the EQ-5D. For the comparison with our data we used the self-reported health of the age group 20–30 years (N = 292, female: 48.3%, mean age: 24.81 ± 3.15 SD, high education (degree or professional qualification): 30.9%; personal communication with Dr. Hinz 17.04.08).

For the comparison of the valuation of hypothetical health states we used the data from the German EQ-5D valuation study by Claes et al. [12], collected in a different random sample of the German population (N = 339, female: 44.8%, ≤ 34 years: 23.0%, high education: 33.0%) [13]. Respondents were asked to value up to 15 different health states from a sample of 43 states. The participants were given selected cards with the description of the health states. These cards had to be ranked on the VAS scale. TTO rating of states was also undertaken. For our comparison we used the collected VAS data.

We used descriptive statistics to describe the sample and health states. Chi-square (χ^2^) statistics were used to test for group differences for nominal data.

## Results

### Sample characteristics

Complete data was available for 161 participants (89.4% participation rate). The mean age of the students was 24.3 (± 4.9 SD) with no significant gender differences (M ± SD male: 24.83 ± 4.86, female: 24.01 ± 4.93; t-Test: p > .05). The majority of the students were female (59.0%). Own illness experience was reported by 25.9% of the sample, 75.3% experienced illness in their close family. As the students have only been in their second year of medical training including no practical training, no more than 49% reported experience of illness in others than close family. No experience with own illness or illness of others reported 13.7% of the sample. About one quarter of the participants were smokers (Table 1).

**Table 1:** Socio-demographics and illness experience (N = 161)

		medical students (N = 161)
Characteristic		

gender (%)^1^	male	39.8
	female	59.0
	missing	1.2

Age		
(mean (SD))		24.34 (4.9)
(Median)		22.5
(min-max)		20–49

Illness Experience (%)	Yourself	25.9
	Family	75.3
	Others	49.1
	none	13.7

Smoker (%)	Never	58.4
	Non-/former smoker	17.4
	Smoker	24.2

Working status (%)	Employed-self/employed	6.4
	Housework	0.6
	Student	91.9
	Other	1.3

Years of education (%)	≤ 9 years	0.0
	10–13 years	82.6
	≥ 14 years	17.4

^1 ^missing data N = 2

The students reported a mean EQ-5D VAS score of 87.3 (± 9.6) with no significant differences between male and female students (male: 87.1 ± 7.8; female: 87.9 ± 10.0, t-Test: p > .05). Compared to a sample of the general population aged 20–30 (M_VAS _= 87.5 ± 14.8 [11]) there were no significant differences (t-Test: p > .05). Male students tended to report lower VAS scores compared to the male general population aged 20–30 (student: 87.1 ± 7.8; general_20–30_: 89.2 ± 13.4, t-Test: p < .10; Table 2). There were no significant differences for females.

**Table 2:** Frequencies for the 5 EQ-5D domains and VAS mean scores – medical students (N = 161) vs. general population aged 20–30 (N = 292) [11]

EQ-5D		medical students	general population aged 20–30 ^2^	chi^2^
mobility (%)	no problems	99.4	97.3	1.30
	moderate problems^1^	0.6	2.7	
	severe problems^1^	-	-	

self-care (%)	no problems	100.0	100.0	^3^
	moderate problems	-	-	
	severe problems	-	-	

daily activities (%)	no problems	96.9	95.2	0.41
	moderate problems^1^	3.1	4.5	
	severe problems^1^	-	0.3	

pain/discomfort (%)	no problems	81.9	89.7	12.1**
	moderate problems^1^	18.1	9.6	
	severe problems^1^	-	0.7	

anxiety/depression (%)	no problems	77.5	89.0	23.7**
	moderate problems	21.9	10.3	
	severe problems	0.6	0.7	

VAS (mean (SD))	male (N = 64^4^/149)	87.1 (7.8)	89.3 (13.4)	-1.50°
	female (N = 95/140)	87.9 (10.0)	85.5 (15.9)	1.42
	total (N = 161/289)	87.3 (9.6)	87.5 (14.8)	-0.17

^1 ^for chi^2^-Test the categories moderate and severe problems have been combined

^2 ^Data from the general population: [11], male/female 20–30 years: personal communication with Dr. Hinz 17.04.08

^3 ^no chi^2^-Test possible

^4 ^gender: missing data N = 2

**p < .01

°p < .10

The students described 10 different EQ-5D health states which, with one exception, were all very mild or mild health states (Table 3). Two-thirds of the participants reported no problems in the 5 areas of the EQ-5D (11111: 65.8%) with a mean VAS score of 89.6 (± 7.0). The health state 11112 (moderately anxious or depressed) was reported by 13.7% of the students, with a mean VAS score of 85.5 (± 9.3). The health state 11121, indicating moderate pain or discomfort, was reported by 9.9% of the students (VAS 82.9 ± 11.1; see other health states in Table 3).

**Table 3:** Self-reported health status on the EQ-5D and mean VAS scores (N = 161)

				VAS	
		
EQ-5D health state		f	%	mean	SD
	11111	106	65.8	89.6	7.00
very mild	11112	22	13.7	85.5	9.34
	11121	16	9.9	82.9	11.1
mild	11122	9	5.6	78.7	11.1
	11123	1	0.6	99.0	-
	11212	3	1.9	92.7	2.5
	11221	1	0.6	90.0	-
	21121	1	0.6	85.0	-
	11222	1	0.6	45.0	-
moderate	21223	1	0.6	50.0	-

	Total	161	100.0	87.3	9.6

The biggest proportion of participants reporting problems in any of the five dimensions was within anxiety/depression (22.5%), with no gender differences (chi^2^-Test: p > .05). No student reported any problems with self-care. Compared to the general population aged 20–30 [11] the students reported significantly more problems in the EQ-5D areas pain/discomfort and anxiety/depression (chi^2^-Test: p < .01; Table 3)

### Valuation of hypothetical health states

The mean VAS scores for the 10 health hypothetical states ranged from 0.815 for the health state 21111 (some problems in walking around) to 0.156 for the health state 32232. Significant gender differences could be found in the VAS valuations for the health states 11113 (male: 0.524 ± 0.170, female: 0.595 ± 0.190) and 12121 (male: 0.658 ± 0.162, female: 0.707 ± 0.147). In both health states the valuations of the female students are significantly higher (t-Test: p < .05).

As there are no valuations of the general population available for Austria, we compared our sample of medical students with valuations of the general population of Germany [12].

There were no significant differences between the VAS scores of medical students and the general population for 7 out of 10 health states including the 2 very mild health states (11112, 21111; t-Test: p > .05). We found significant differences (t-Test: p < .01) for the following 3 health states: 11113 (extremely anxious or depressed), 21323 (severe problems with performing usual activities and extremely anxious or depressed, some problems walking around and moderate pain or discomfort) and 32232 (confined to bed and extreme pain/discomfort, some problems in washing and dressing myself, some problems in performing usual activities and moderately anxious or depressed). In all 3 health states the medical students valued the hypothetical health states higher than the general population. For the health state 12121 there was a tendency (p < .10) towards a higher valuation by the medical students (Table 4).

**Table 4:** Comparison of VAS scores of medical students (N = 161) and the general population (N = 339) for 10 hypothetical health states

		medical students		general population^1^		
		
		M^rank^	SD	M^rank^	SD	t (df = 498)
very mild	21111	0.815 ^1^	0.108	0.82 ^1^	0.13	-0.45
	11112	0.810 ^2^	0.129	0.80 ^2^	0.14	0.79
mild	12121	0.695 ^3^	0.145	0.67 ^3^	0.17	1.7°
	11113	0.571 ^4^	0.181	0.51 ^5^	0.25	3.10**
moderate	12222	0.549 ^5^	0.151	0.54 ^4^	0.18	0.58
	21232	0.384 ^6^	0.165	0.37 ^6^	0.20	0.83
	21323	0.354 ^7^	0.145	0.30 ^7^	0.16	3.76**
severe	22323	0.273 ^8^	0.139	0.25 ^8^	0.16	1.65
	32223	0.157 ^9^	0.106	0.16 ^9^	0.11	-0.29
	32232	0.156 ^10^	0.109	0.13 ^10^	0.09	2.63**

^1 ^[12]

** p < .01

°p < .10

## Discussion

Medical decision-making relies heavily on the value attached to a specific health state by patients, health care professionals or the general public. Risky procedures are usually undertaken in order to obtain relief from very poor health states. However, the assessment of risk and the value of potential benefits are not usually made explicit and are difficult to communicate. Medical students might have a different perception of health and therefore value health states differently compared to the general population.

In this study we describe how medical students value hypothetical health states in comparison to the general population. In the valuation process the future doctors had to take on a different perspective on health, namely the side of someone who is actually suffering and in need for help. The students were confronted with the question of "how would I feel and how would I decide about medical interventions if I were in a particular health state".

The comparison of our data on health state valuation by medical students with the results for the general population [12] showed significant differences for 3 of 10 health states including one mild, one moderate and one severe health state, in other words every third health state is valued differently independent of the level of severity of the health state. Overall the results show that if medical students value health states differently, they value them higher than the general population. However, on the basis of our data we can not attribute these differences only due to experiences and gained knowledge of the students during first year in medical school. Socioeconomic background or high level of education are potential confounding variables as medical students are a highly selected group.

Medical students rated their own health as very good with no significant differences to the general population aged 20–30 [11] on the EQ-5D VAS. In regard to the single EQ-5D areas, medical students report significantly more problems concerning pain/discomfort and anxiety/depression compared to the general population aged 20–30. These results are partly supported by previous findings of a higher prevalence for anxiety and depression in medical students [14,15] and a deterioration in vitality and increased difficulty carrying out daily activities because of physical or emotional problems over a 10 months period of medical students in their final year [16].

## Conclusion

Based on our results we can conclude that medical students have the tendency to value health states higher than the general public. Medical professionals should be continuously aware that their assessment of a particular health state can differ from the valuations of the general population. Therefore it is important to collect patients individual assessment of their own health status and to integrate this value in the decision making process by means of standard HRQL instruments.

Futures studies should investigate the change of health states valuations of health care professionals over the period of their medical training.

## Competing interests

The authors declare that they have no competing interests.

## Authors' contributions

MTB and SH drafted the manuscript. BN and DR organized and carried out the original study. MTB performed the statistical analysis. GR and SH designed the study protocol. All authors read and approved the final manuscript.
